# *NBAS*, a gene involved in cytotoxic degranulation, is recurrently mutated in pediatric hemophagocytic lymphohistiocytosis

**DOI:** 10.1186/s13045-022-01318-z

**Published:** 2022-07-28

**Authors:** Xiaoman Bi, Qing Zhang, Lei Chen, Dan Liu, Yueying Li, Xiaoxi Zhao, Ya Zhang, Liping Zhang, Jingkun Liu, Chaoyi Wu, Zhigang Li, Yunze Zhao, Honghao Ma, Gang Huang, Xin Liu, Qian-fei Wang, Rui Zhang

**Affiliations:** 1 CAS Key Laboratory of Genomic and Precision Medicine, Beijing Institute of Genomics, Chinese Academy of Sciences, Beijing, 100101 China; 2 Hematologic Disease Laboratory, Hematology Center, Beijing Key Laboratory of Pediatric Hematology Oncology, National Key Discipline of Pediatrics (Capital Medical University), Key Laboratory of Major Diseases in Children, Ministry of Education, Beijing Pediatric Research Institute, Beijing Children’s Hospital, Capital Medical University, National Center for Children’s Health, Beijing, 100045 China; 3 China National Center for Bioinformation, Beijing, 100045 China; 4 University of Chinese Academy of Sciences, Beijing, 100049 China; 5 Hematology Center, Beijing Key Laboratory of Pediatric Hematology Oncology, National Key Discipline of Pediatrics (Capital Medical University), Key Laboratory of Major Diseases in Children, Ministry of Education, Beijing Children’s Hospital, Capital Medical University, National Center for Children’s Health, Beijing, 100045 China; 6 Divisions of Pathology and Experimental Hematology and Cancer Biology, Cincinnati Children’s Hospital Medical Center, Cincinnati, OH 45229 USA; 7 Key Laboratory of Tropical Translational Medicine of Ministry of Education, College of Biomedical Information and Engineering, Hainan Medical University, Haikou, 571199 China

**Keywords:** Hemophagocytic lymphohistiocytosis, Germline variants, Trios, *NBAS*, NK-cell

## Abstract

Hemophagocytic lymphohistiocytosis (HLH), particularly primary HLH (pHLH), is a rare, life-threatening disease. Germline genetic deficiency of 12 known HLH genes impairs cytotoxic degranulation in natural killer (NK) cells or cytotoxic T lymphocytes (CTLs) and contributes to pHLH development. However, no pathogenic mutations in these HLH genes are found in nearly 10% of HLH patients, despite a strong suspicion of pHLH, suggesting that the underlying genetic basis of HLH is still unclear. To discover novel susceptibility genes, we first selected 13 children with ppHLH (presumed primary HLH patients in the absence of detectable known HLH gene variants) and their parents for initial screening. Whole-genome sequencing (WGS) in one trio and whole-exome sequencing (WES) in twelve trios revealed that two ppHLH patients carried biallelic *NBAS* variants, a gene that is involved in Golgi-to-endoplasmic reticulum (ER) retrograde transport upstream of the degranulation pathway. Additionally, two candidate genes, *RAB9B* and *KLC3*, showed a direct relationship with known HLH genes in protein-protein interaction (PPI) network analysis. We analyzed *NBAS*, *RAB9B*, *KLC3* and known HLH genes in an independent validation cohort of 224 pediatric HLH patients. Only biallelic *NBAS* variants were identified in three patients who harbored no pathogenic variants in any of the known HLH genes. Functionally, impaired NK-cell cytotoxicity and degranulation were revealed in both *NBAS* biallelic variant patients and in an *NBAS*-deficient NK-cell line. Knockdown of NBAS in an NK-cell line (IMC-1) using short hairpin RNA (shRNA) resulted in loss of lytic granule polarization and a decreased number of cytotoxic vesicles near the Golgi apparatus. According to our findings, *NBAS* is the second most frequently mutated gene (2.11%) in our HLH cohort after *PRF1*. NBAS deficiency may contribute to the development of HLH via a dysregulated lytic vesicle transport pathway.

**Supplementary Information:**

The online version contains supplementary material available at 10.1186/s13045-022-01318-z.

## To the Editor,

Hemophagocytic lymphohistiocytosis is a rare syndrome characterized by systemic inflammation, hypercytokinemia and multiorgan failure [1]. Primary HLH typically occurs in early childhood and is caused by pathogenic variants in 12 known HLH genes [1, 2]. Allogeneic hematopoietic stem cell transplantation (HSCT) remains the only definitive curative therapy for pHLH [3, 4]. According to the HLH-2004 protocol, variants in HLH genes serve as independent diagnostic criteria for pHLH and a guide to help select treatment options [5]. However, in nearly ten percent of patients clinically strongly suggested to have pHLH, no pathogenic variants in known HLH genes are present [6, 7].

**Fig. 1:**
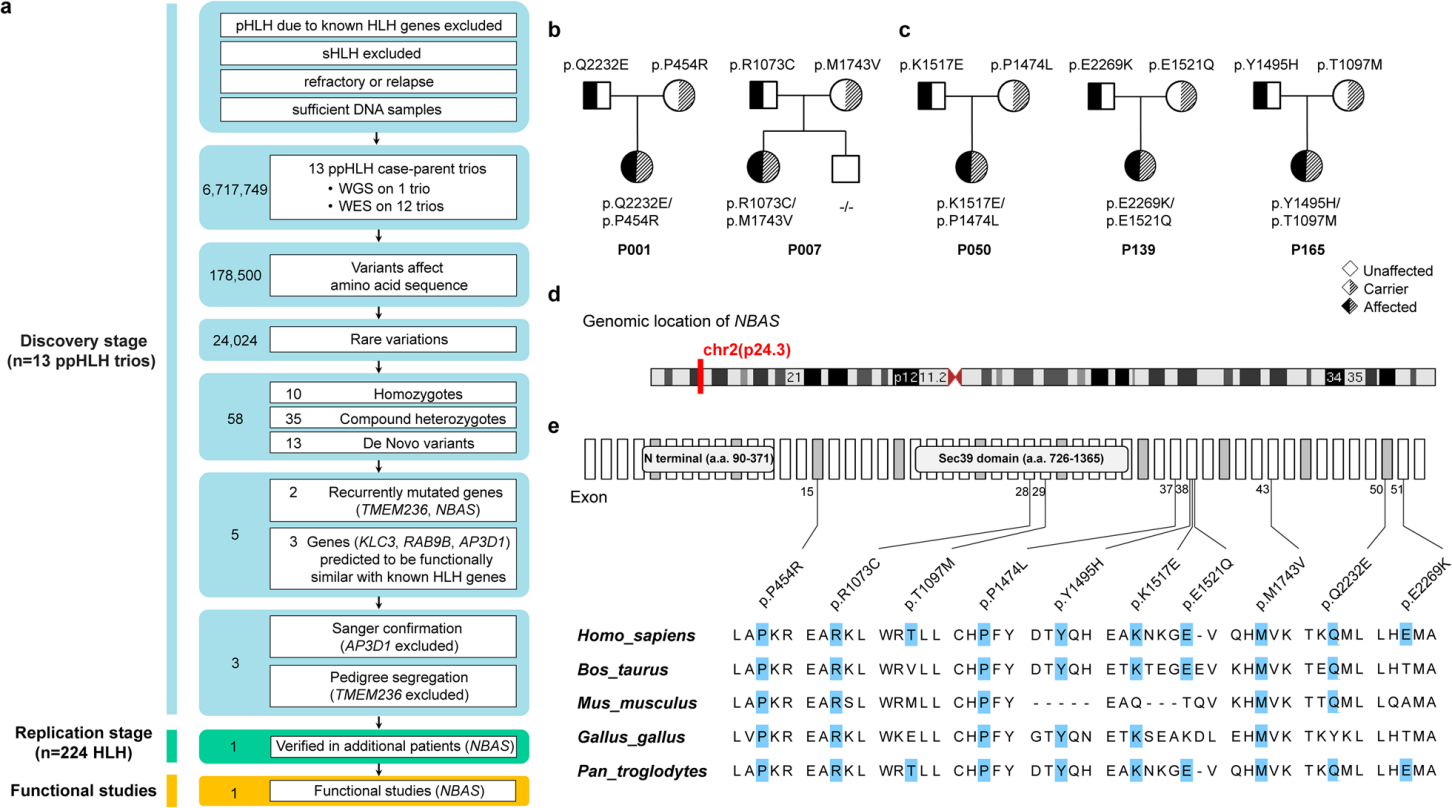
Identification and bioinformatic characterization of *NBAS* biallelic variants. **a** Schematic representation of gene prioritization and validation strategies applied in this study. *NBAS* genotypes of two ppHLH families in the discovery stage (**b**) and three families in the replication stage (**c**). Closed symbols indicate affected patients, and open symbols indicate unaffected family members. **d** Schematic diagrams of the genomic location of *NBAS*. **e** Distribution of NBAS variants identified in this study (top) and the evolutionary conservation of mutated amino acids in the NBAS protein among different species (bottom). All 52 exons of the *NBAS* gene (reference sequence NM_015909) and two known protein domains of the NBAS protein are represented. ppHLH, presumed primary HLH; WES, whole-exome sequencing; WGS, whole-genome sequencing

In this study, we performed WES (n = 12) or WGS (n = 1) in 13 ppHLH parent-child trios (strongly suspected pHLH cases despite lacking a confirmed genetic diagnosis). A total of 6,717,749 variants were successfully called across the 13 ppHLH patients (Fig. 1a). After filtering, we identified 58 genotypes that may contribute to HLH (Additional file 1: Methods and Fig. S1, Additional file 2: Table S1). The majority of the genes annotated by these variants appeared to be patient specific, except for *TMEM236* and *NBAS*. In PPI network analysis, three genes (*RAB9B*, *KLC3* and *AP3D1*) showed molecular relationships with known HLH genes (Additional file 1: Fig. S2). Finally, only the recurrently mutated gene *NBAS* and two genes (*RAB9B* and *KLC3*) from the PPI network remained after Sanger confirmation and pedigree segregation analysis (Fig. 1b and Additional file 1: Fig. S3).

To examine whether variants in the three candidate genes are present in other HLH patients, we extended our genetic study to a validation cohort of 224 pediatric HLH patients (Additional file 2: Tables S2 and S3). Variants in only *NBAS* were found in three additional HLH children, with confirmation by Sanger sequencing in a family setting (Fig. 1c). Collectively, a total of five patients in our study carried *NBAS* biallelic variants (Fig. 1d–e and Additional file 2: Table S4). The estimated frequency of *NBAS* variants among the pediatric HLH patients was 2.11%, which is lower than that of *PRF1* but higher than that of the other 11 known pHLH genes (Additional file 2: Tables S5 and S6). The main clinical characteristics at baseline of all pHLH patients, including the 5 with *NBAS* variants, are summarized in Additional file 2: Table S7.

**Fig. 2:**
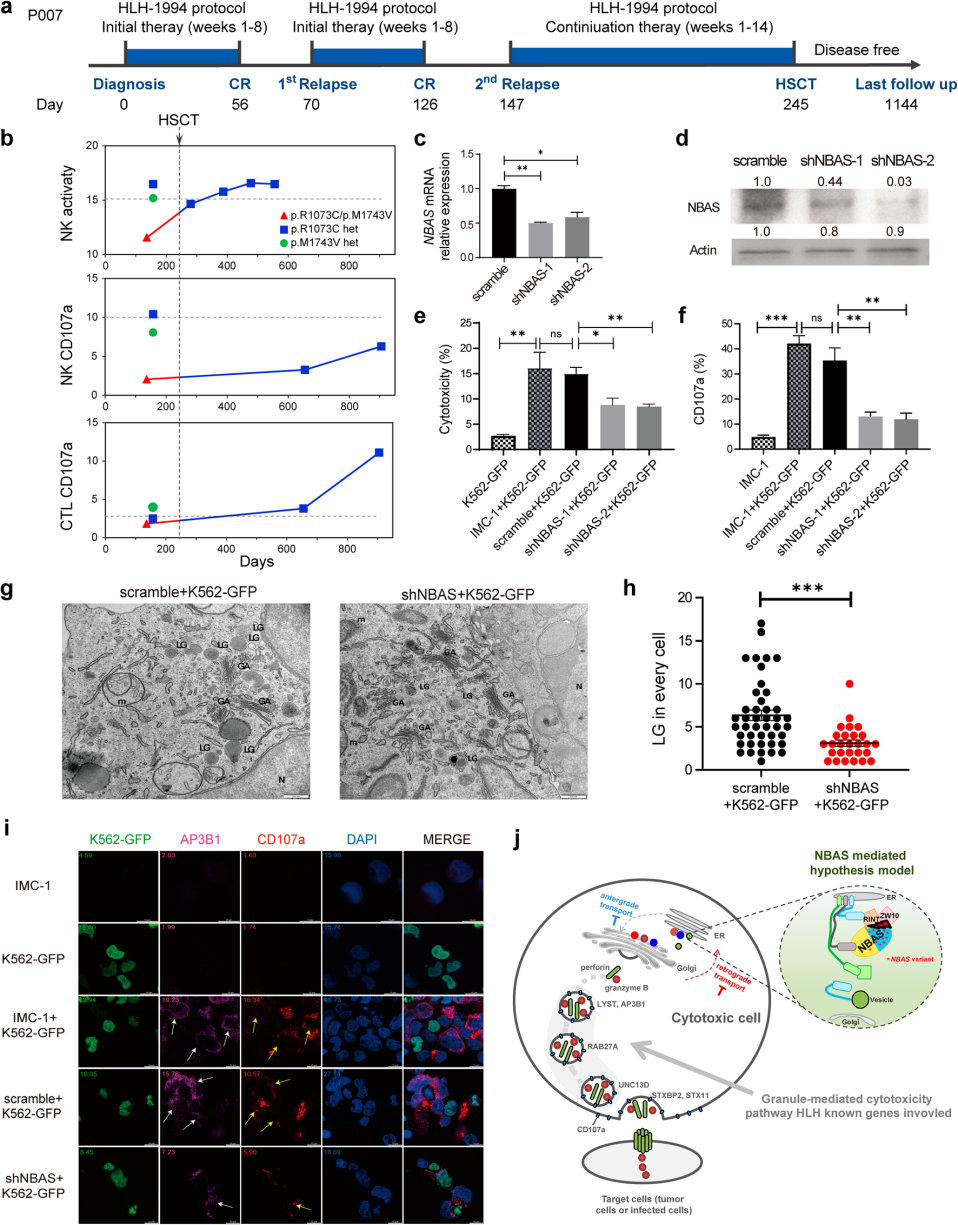
NBAS is required for cytotoxic granulation in the NK-cell line. **a** Plot showing the HLH time course, therapeutic approaches and response status in patient P007 with presumed primary HLH. **b** Functional investigations of cytotoxic lymphocytes. NK-cell cytotoxicity (upper row) and NK (middle row) and T-cell degranulation (bottom row) were defective in P007 compared with her healthy parents but gradually recovered after HSCT. NBAS mRNA (**c**) and protein (**d**) levels in an NK-cell line (IMC-1) after NBAS knockdown (n = 3). **e** Histograms showing the cytotoxic activity of scramble or shNBAS-targeting IMC-1 cells analyzed by FACS after coculture with K562-GFP target cells (n = 3). K562-GFP was used as the negative control, and K562-GFP cocultured with wide-type IMC-1 (without transfection of any shRNA) was used as the positive control. **f** Histograms show surface CD107a expression, indicating the degranulation ability of scramble or shNBAS-targeting IMC-1 cells in the presence of K562-GFP target cells. **g** Representative electron microscopic images of sorted scramble or shNBAS IMC-1 cells stimulated with K562-GFP cells for 4 h. LG, lytic (cytotoxic) granule; N, nucleus; M, mitochondria; GA, Golgi apparatus. Scale bars = 500 nm. **h** The relative number of lytic granules per field was quantified (n = 3). **i** Representative images showing decreased expression of AP3B1 (magenta; indicated by white arrows) and CD107a (red; indicated by yellow arrows) stimulated by K562-GFP (green) cells for 4 h. Nuclei were stained with DAPI (blue). Scale bar: 10 μm. n = 3. **j** Schematic depicting the process by which cytotoxic cells kill target cells through the granule-mediated degranulation pathway (left panel). The pop-up panel shows a cartoon of NBAS, along with RINT and ZW10, as part of the syntaxin 18 complex between the endoplasmic reticulum (ER) and Golgi. Data were presented as means ± SEM. *P < 0.05; **P < 0.01; ***P < 0.001; ns, not significant

To explore the functional relevance of *NBAS* variants in HLH, we focused on patient P007, who presented with recurrent HLH as soon as therapy was discontinued after complete remission (Fig. 2a). Prompted by a suspicion of pHLH, functional investigations were performed after her second relapse. For both NK cells and CTLs from P007, cytotoxic function and degranulation were defective compared with those of her healthy parents (heterozygous carriers of *NBAS*); however, progressive recovery in these functions occurred after she received haploidentical HSCT from her father (Fig. 2b). This is consistent with the notion that HLH gene variants reported to date are generally loss-of-function. Therefore, we performed RNA interference of *NBAS* by using two different shRNAs in the NK-cell line IMC-1, and the *NBAS* shRNA-targeted (shNBAS) cells showed impaired cytotoxicity and degranulation (Fig. 2c–f and Additional file 1: Figs. S4 and S5), consistent with the abnormalities in the cells from patients.

Considering that NBAS is known to be essential for NK-cell cytolytic function and Golgi-to-ER retrograde transport [8–11], NBAS may function upstream of the lymphocyte degranulation process. As shown in Fig. 2g–h, knockdown of *NBAS* decreased the number of cytotoxic vesicles, particularly near the Golgi apparatus. Furthermore, shNBAS IMC-1 cells exhibited significantly decreased expression of AP3B1, an important protein upstream of the known degranulation pathway involved in the transport of cytotoxic vesicles from the Golgi (Fig. 2i and Additional file 1: Fig. S6). Taken together, these findings suggest that *NBAS* defects disrupt the transport and recycling of proteins or vesicles between the ER and Golgi apparatus and then impact the downstream cytotoxic vesicle transport and degranulation cascades involved in HLH (Fig. 2j).

Biallelic mutations in *NBAS* have been related to a wide spectrum of symptoms, whereas mutations occurring at Sec39 domain and C-terminus mainly associated with liver failure and multisystemic features, respectively [12]. *NBAS* mutated HLH in this study was not associated with these clinical manifestations, and 90% of mutations identified clustered within the latter region of the Sec39 and C-terminal domains. It remains to be determined whether differences in mutation spectrum contribute to differential phenotypic manifestations. Collectively, our data provide compelling evidence that the recurrent mutated gene *NBAS*, known for being involved in transport between the Golgi and ER, is an HLH-predisposing gene that may play a role upstream of the known degranulation pathway in NK cells.

## Supplementary Information

**Additional file 1:** Methods and supplementary figures S1 to S6.

**Additional file 2:** Supplementary tables S1 to S7.

## Data Availability

The datasets generated and analyzed during the current study are available in the Genome Sequence Archive in National Genomics Data Center, Beijing Institute of Genomics (China National Center for Bioinformation), Chinese Academy of Sciences, under accession number HRA000101 that is publicly accessible at https://bigd.big.ac.cn/gsa-human/. All data generated or analyzed during this study are included in this published article and its supplementary information files.

## Abbreviations

CTLs: Cytotoxic T lymphocytes
ER: Endoplasmic reticulum
HSCT: Hematopoietic stem cell transplantation
HLH: Hemophagocytic lymphohistiocytosis
NK: Natural killer
ppHLH: Presumed primary HLH
pHLH: Primary HLH
PPI: Protein-protein interaction
shRNA: Short hairpin RNA
WES: Whole-exome sequencing
WGS: Whole-genome sequencing
