# Supplementary material for: NBAS, a gene involved in cytotoxic degranulation, is recurrently mutated in pediatric hemophagocytic lymphohistiocytosis
Source: J Hematol Oncol. 2022 Jul 28;15:101. doi: 10.1186/s13045-022-01318-z (PMC9331571; doi:10.1186/s13045-022-01318-z)
Supplement: Supplementary file 1 — Additional file 1: Methods and supplementary figures S1 to S6. [file 13045_2022_1318_MOESM1_ESM.docx]

**Additional file 1 for**

***NBAS*, a gene involved in cytotoxic degranulation, is recurrently mutated in pediatric hemophagocytic lymphohistiocytosis**

Xiaoman Bi^1,3,4,7^†, Qing Zhang^2^†, Lei Chen^1,3,4^†, Dan Liu^1,3^, Yueying Li^1,3^, Xiaoxi Zhao^2^, Ya Zhang^1,3,4^, Liping Zhang^5^, Jingkun Liu^1,3,4^, Chaoyi Wu^1,3,4^, Zhigang Li^2^, Yunze Zhao^5^, Honghao Ma^5^, Gang Huang^6^, Xin Liu^1,3,4^*, Qian-fei Wang^1,3,4^*, Rui Zhang^5^*

* Corresponding authors. Email: liuxin@big.ac.cn (X.L.); wangqf@big.ac.cn (Q.W.); ruizh1973@126.com (R.Z.).

This file includes:

Materials and Methods

Supplementary figures S1 to S6

**Materials and Methods**

**Study populations**

This study included 237 unrelated children who were diagnosed as HLH according to HLH-2004 criteria[1] at Beijing Children’s Hospital from June 2014 to November 2018. We also encouraged the parents and unaffected siblings to participate in the study. Clinical outcomes and examinations, including HLH gene panel testing and clinical WES tests, were extracted from their medical record. We selected 13 complete trios (HLH patient and his/her parents) for initial screening of HLH novel causal genes. Our selection criteria include: (i) patients have no biallelic variants in 12 known HLH genes and, (ii) patients have no malignant, autoimmune or autoinflammatory diseases and, (iii) patients have a positive refractory or relapse history before April 2017, and (iv) the DNA amount of all three persons meet the minimum requirement for WES (1ug) or WGS (20ng). We consider these 13 patients as ppHLH. Those HLH children who were not in the discovery stage were considered as replication samples (n=224). Informed consent was obtained from all study participants. The study was approved by the Institutional Review Board of Beijing Institute of Genomics (China National Center for Bioinformation).

**WES and WGS**

Genomic DNA was extracted from peripheral blood or bone marrow from study participants using the QIAamp DNA Blood Mini Kit (Qiagen) according to the manufacturer’s protocol. Whole-genome sequencing (～30X average depth) was performed on one trio due to the insufficient quantity of their DNA samples for exome capture. Whole-exome sequencing (～100X average depth) was performed on 36 samples, including 12 parent-child trios. Exomes were captured using Agilent SureSelect V6 kits according to the manufacturer’s instructions. Paired-end sequencing was performed on an Illumina Hiseq X ten with 150bp read length. Both research WES (n=36) and WGS (n=3) were conducted by Novogene company, while clinical (n=37) WES was conducted by MyGenostics company.

**Targeted sequencing**

To examine the recurrence of the most promising candidate genes identified from initial screening, targeted next generation sequencing (NGS) was performed in a subset of the replication sample who had no clinical WES data (n=187). A targeted panel also covering 12 known HLH genes was designed according to CleanPlex technology (Paragon Genomics, CA, USA). Sequencing was performed using established protocols on an Illumina HiSeq X Ten system. The effective coverage of our initial target sequences was estimated to be 99.52% of the initial regions of interest. All the samples achieved at least 90% of the target bases covered to at least a depth of 10X.

**Variant calling and filtering**

SNV/indel calling and annotation were analyzed for germline variants following the stepwise procedures of the Genome Analysis Toolkit (GATK) Best Practices pipeline. Briefly, read pairs were aligned to the human reference genome (hg38) by Burrow-Wheeler Aligner. Mapped reads were further refined using Picard program suites and GATK, which included removing PCR duplicate reads and realigning reads around potential indel regions. GATK Haplotype Caller was applied to call both SNVs and indels, followed by ANNOVAR to perform variant functional annotation. In silico methods (SIFT[2], PolyPhen[3] and CADD[2]) were used to predict the impact of amino acid substitutions on proteins.

All the variants detected were subsequently filtered by a homemade pipeline, excluding (i) variants with a read depth (DP) less than 10 for samples that underwent WES or target sequencing, or less than 6 for samples that underwent WGS; (ii) variants with strand bias (covered by only forward or reverse reads); (iii) variants present within intergenic and intronic regions and synonymous variants; (iv) variants with minor allele frequency (MAF) greater than 1% in east Asian populations included in the internal cohort[4] or public databases (dbSNP, 1000 Genomes, EXAC and gnomAD); (v) homozygous genotypes with an alt-ratio less than 0.3 and heterozygous genotypes with an alt-ratio greater than 0.7 or less than 0.3. We assume that the penetrance of carrying two HLH causal variants is 100% and further excluded: (vi) homozygotes reported in above east Asian populations and 26 healthy parents of 13 trios; compound heterozygotes reported or either of the two variants that occurred in a homozygous state in above east Asian populations and 26 healthy parents of 13 trios. We also excluded de novo variants reported in the same general populations. The detailed procedures of the pipeline are illustrated in Fig. 1a.

**Protein-protein interaction (PPI) network analysis**

To prioritize unknown HLH-related candidate genes, a network proximity analysis was used to screen for additional candidate genes that may biologically related to the known HLH genes[5-7]. We performed Ingenuity Pathway Analysis (IPA) based protein network analysis using all possible candidate genes identified from initial screening in 13 ppHLH trios and 12 HLH known genes. The reference set of IPA was based on the high-quality manual curation of texts from published journals and public databases. Both direct and indirect relationships between molecules based on experimentally observed data were included. To obtain strongest interactions, only nearest neighbors of HLH known genes based upon network proximity were retained. We further confirmed all the relationship by manually review in retrieved references. The network was further optimized to improve graphical representation using Cytoscape software[8].

**Sanger sequencing**

We conducted experimental validation using Sanger sequencing for the variants in the most promising genes identified from initial WGS/WES screening and those in recurrent genes confirmed after WES/target sequencing analyses. We included the parents and unaffected siblings of the variants' carriers into the validation test if their DNA samples were available. Information regarding primers and PCR conditions is available upon request. The PCR products were purified and inserted into pEASY-Blunt vector. E. coli carrying plasmid was grown at 37℃ in LB medium with 100 μg/mL ampicillin. The white clones were screened using blue-white screening. Next, the vectors were bidirectionally sequenced on an ABI 3730XL DNA Analyzer (Applied Biosystems).

**Immunological analyses of HLH patients**

Nature killer (NK) cell cytotoxicity and intracellular expression of CD107a were evaluated by flow cytometry using peripheral blood mononuclear cells (PBMCs) from patients. All flow cytometry data were acquired on a LSR Fortessa instrument (FACSCalibur flow cytometer, BD Biosciences, USA).

**Lentiviral preparation**

HEK293T cells were co-transfected with commercial human shRNA expression lentiviral vectors (Sigma) and packaging plasmids (pMD2.G and psPAX2) using the FuGENE HD transfection reagent (Promega E2311). Supernatants were collected 48 hours and 72 hours after transfected and concentrated 100-fold by PEG 8000 precipitation (Solarbio, P8260). The lentivirus stocks were aliquoted and kept frozen at -80℃.

**Cell transfection**

IMC-1 cells were cultured in RPMI-1640 in the presence of FBS (20%) and IL-2 (10 ng/mL) and seeded in 12-well plates (1×10^6^ cells/well) transduced with lentiviral particles for 12h followed by a second transduction. After an additional 24 hours, cells were selected with puromycin (Amresco, J593).

**Cytotoxicity assays**

The target cells K562 erythroleukemia expresses high levels of green fluorescent protein (K562-GFP). The lentiviral transduced IMC-1 cells were mixed with K562-GFP cells at a 4:1 effector to target (IMC-1: K562-GFP) ratio for 4 hours at 37℃ in 5% CO_2_. After incubation for 4 hours, cells were then stained with live/dead near-IR dye (Invitrogen) and analyzed by flow cytometry (Bechman, Moflo XDP) with FlowJo software. The compromised K562-GFP cells target cells were stained with live/dead near-IR dye and showed dual (green-red) fluorescent[9, 10].

**Degranulation assays**

The transduced IMC-1 cells were mixed with K562-GFP cells at a 1:1 effector to target (IMC-1: K562-GFP) ratio, incubated 4 hours at 37℃ in 5% CO_2_ and surface expression of CD107a were evaluated by flow cytometry. In brief, cells were harvested, washed with PBS, surface stained with eFluor 660 conjugated anti-CD107a (LAMP-1) mAb (IgG1, kappa; eBioscience™) for 30 min at 4°C and analyzed by flow cytometry with FlowJo software[11].

**Transmission electron microscopy**

The transduced IMC-1 cells were mixed with K562-GFP cells at a 1:1 effector to target (IMC-1: K562-GFP) ratio and incubated 4 hours at 37℃ in 5% CO_2_. And at least 1×10^6^ IMC-1 cells were sorted by flow cytometry and immediately fixed with 2.5% glutaraldehyde at 4 ℃ overnight. Electron microscopy was performed at the Center for Biological Imaging of the Chinese Academy of Sciences, and observed by transmission electron microscopy (Talos L120C 120 kv).

**Immunofluorescence**

Cells were washed with PBS and fixed using 4% paraformaldehyde solution in PBS, followed by permeabilization with 0.5% Triton X-100, blocked in 0.5% BSA. Cells were incubated with primary antibodies overnight at 4 ℃. The primary antibodies as follows: Rabbit anti-human AP3B1 (Novus), mouse anti-human CD107a conjugated with eFluor 660 (eBioscience). After three rounds of washing using PBS, cells were incubated in secondary antibody for1 hour. Secondary antibody was goat antibodies specific for rabbit primary antibodies, conjugated with Alexa Fluor 555 (Invitrogen). DNA was stained with DAPI (Vector). Images were collected with Leica SP8 microscope.

**Image acquisition and analysis**

Images of the control and experimental groups were acquired under identical imaging conditions on the same microscope. Image acquisition was performed using a confocal microscope (Leica TCS-SP8) either with 63×/1.32 numeric aperture (NA) oil objective. Four laser excitations were used (405 nm, 488 nm, 532 nm, 633 nm for DAPI, FITC, Alexa dyes 555 and APC, respectively). Image analysis was performed with Leica LAS X software (https://www.leica- microsystems.com/products/microscope-software/p/leica-las-x-ls/). The intensity of an equivalent ROI in the cytoplasm of the same cell was subtracted to correct for cytoplasmic background.

**Statistical analysis**

Two-tailed Wilcoxon rank sum test was used to estimate the statistical significance of age of HLH patients between discovery cohort and validation cohort. Fisher's exact test was used to evaluate the frequency of monoallelic variants in genes between our HLH cohort and 1000 Genomes Project. P values less than 0.05 were considered statistically significant. The data presented in experimental analysis are the result of at least 3 independent experiments (n≥3). Statistical analysis was performed using GraphPad Prism (version 7, San Diego, CA). Unpaired t-test and a p<0.05 was considered statistically significant.

**Data availability**

All sequence data reported in this paper have been deposited in the Genome Sequence Archive[12] in National Genomics Data Center[13], Beijing Institute of Genomics (China National Center for Bioinformation), Chinese Academy of Sciences, under accession number HRA000101 that is publicly accessible at <https://bigd.big.ac.cn/gsa-human/>.

**Supplementary Figures:**


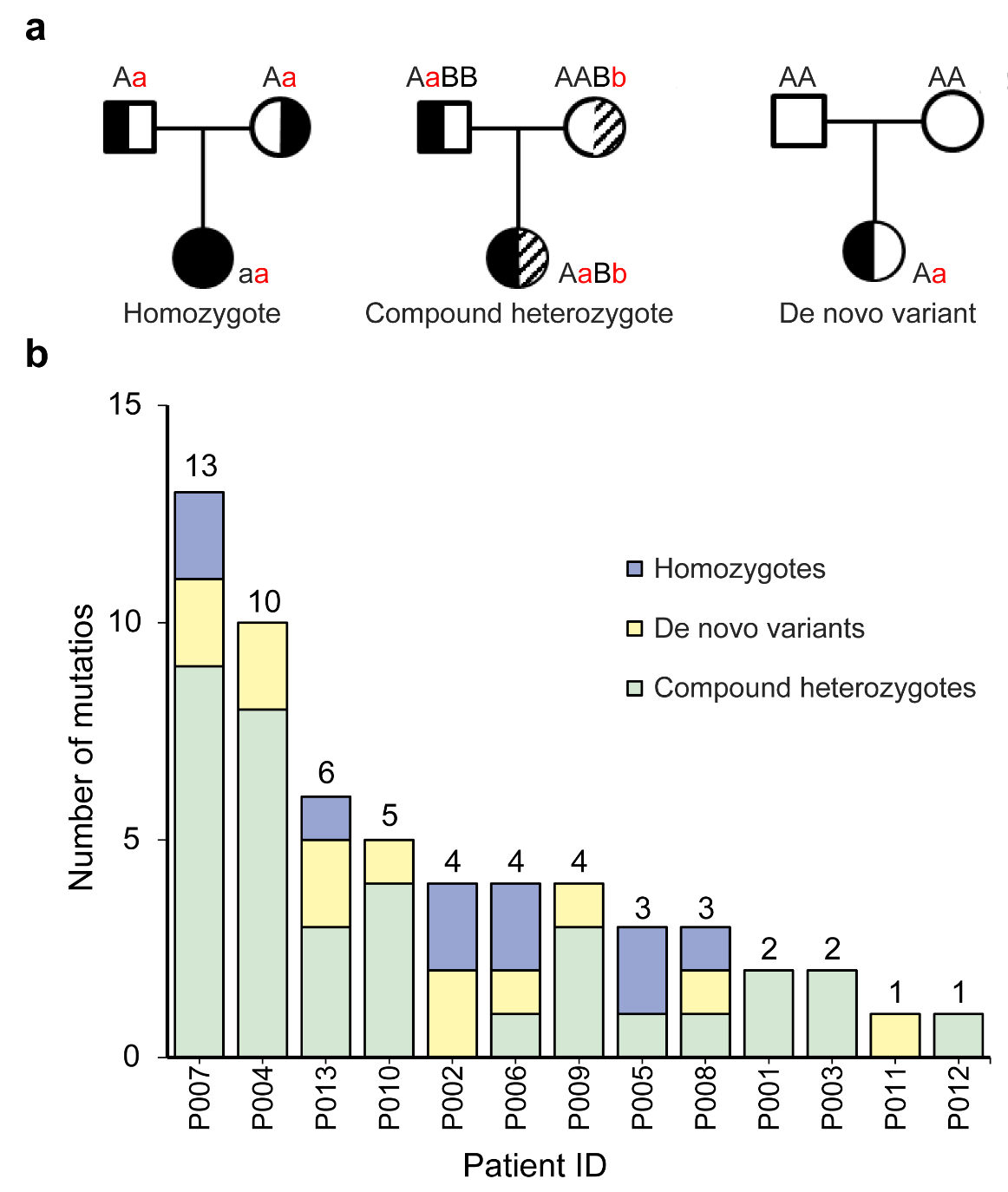


**Fig. S1. Germline variants identified in 13 ppHLH trios. a** Three types of germline variants collected in this study using rare-disease inheritance models. **b** Number and type of non-silent germline variants in 13 ppHLH patients.


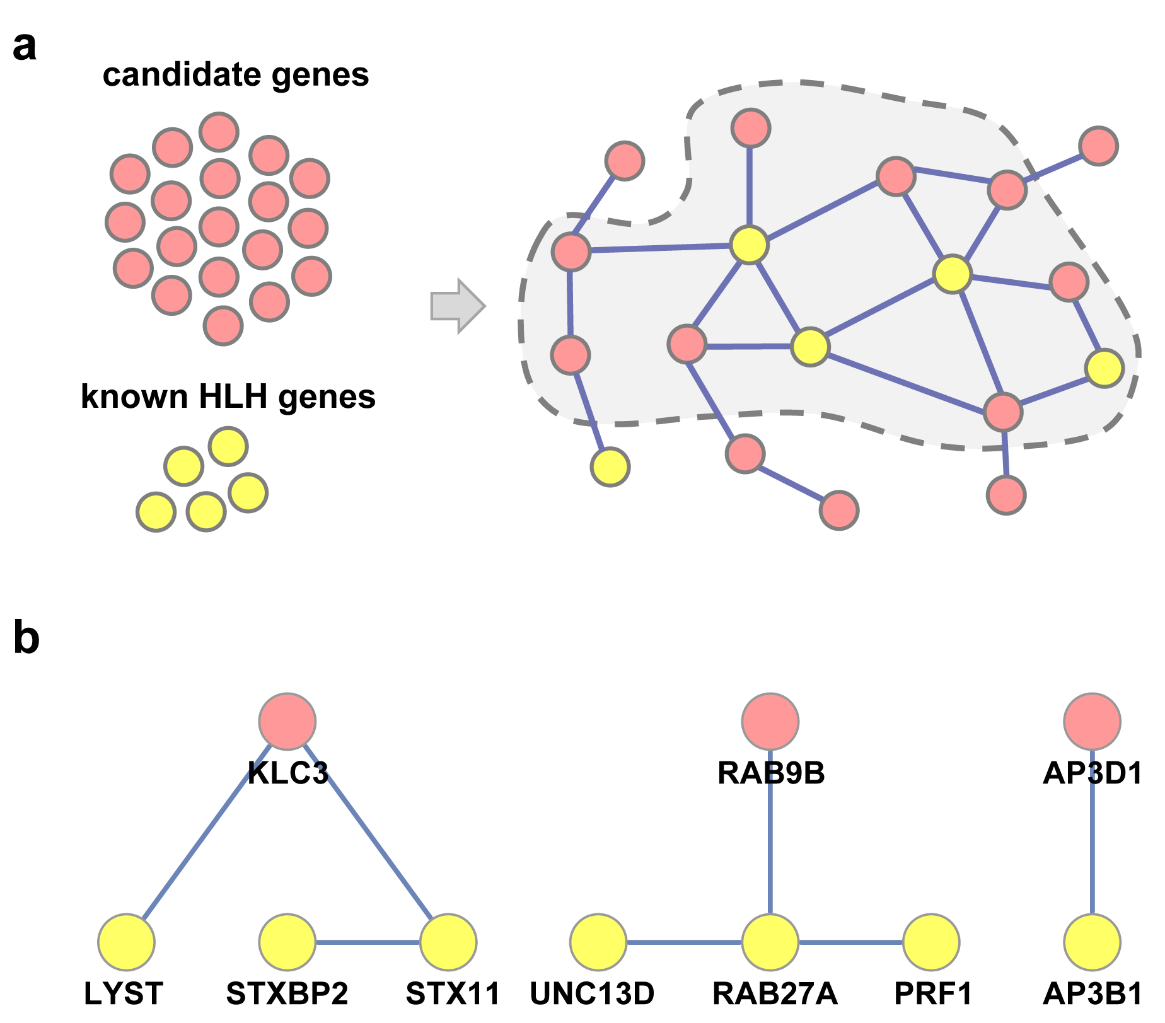


**Fig. S2. Identification of HLH-associated related genes in network analysis.** Prioritizing schemes for finding HLH-associated candidates. Candidates and known HLH genes are mapped to interactome networks. Only nearest neighbors of HLH known genes based upon network proximity were retained. **b** The PPI network contains 3 HLH candidate genes and 7 HLH known genes in our study. Genes are represented as nodes and the biological relationship between two nodes is represented as an edge.


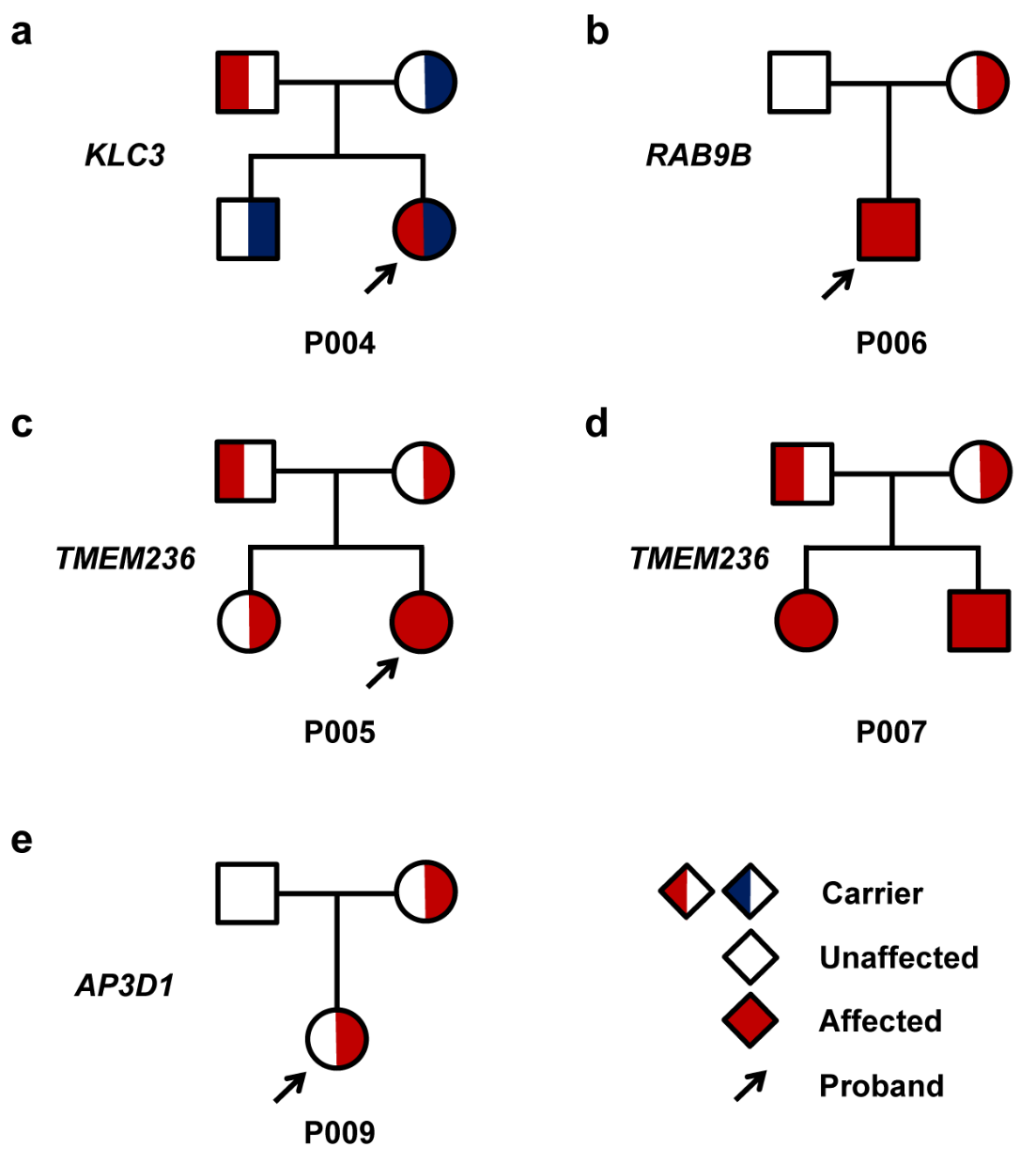


**Fig. S3. Pedigrees of patients with promising variants.** Filled symbols represent affected individuals, half-filled symbols indicate unaffected carriers, and open symbols represent unaffected members. The arrow indicates the proband.


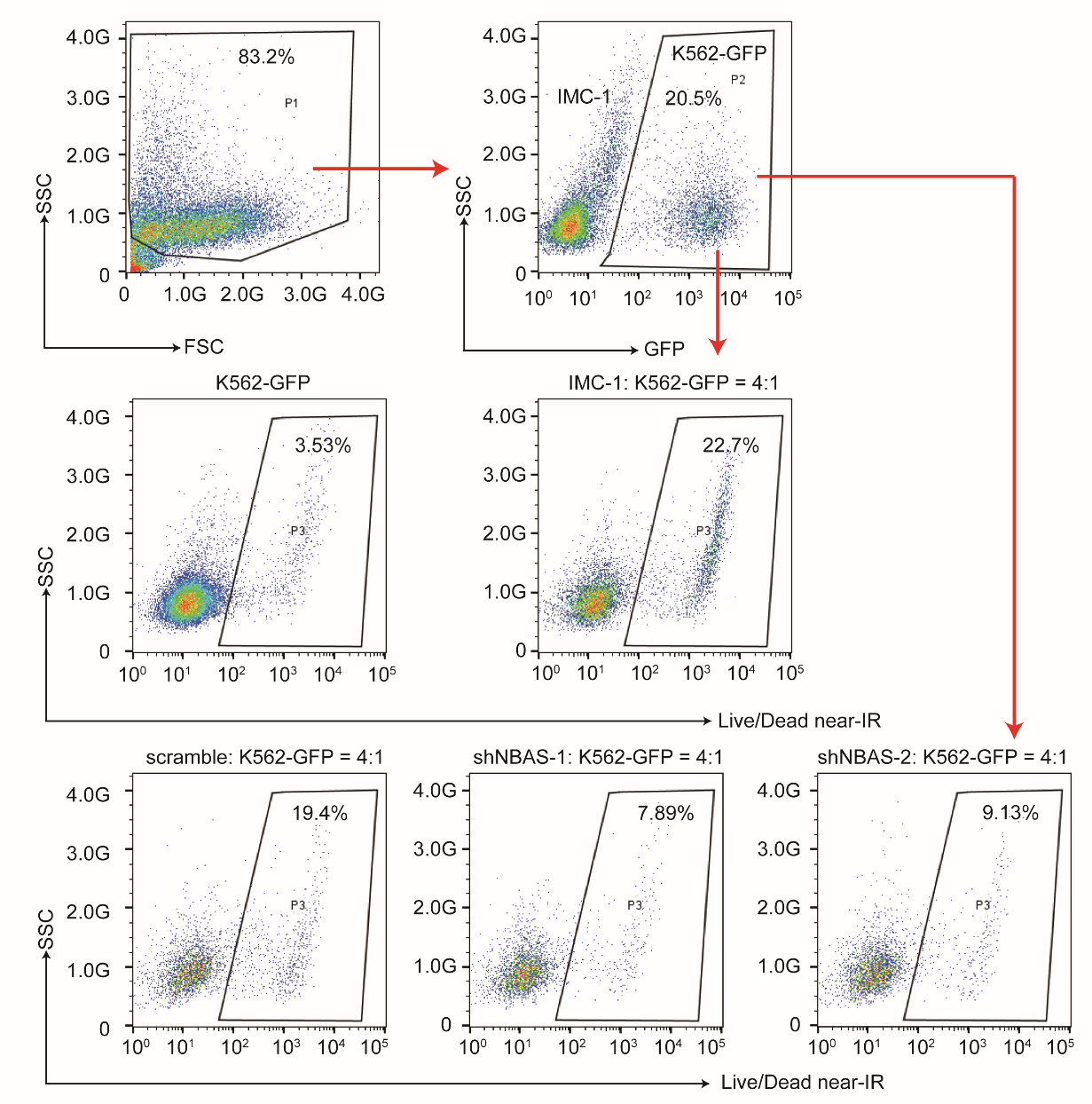


**Fig. S4. Knockdown NBAS impairs the cytotoxic activity in NK cell line.** Flow cytometric analysis of cytotoxic activity indicated by the dead K562-GFP cells. K562-GFP target cells were co-cultured with scramble or shNBAS IMC-1 effector cells for 4 hours. The effector-to-target (E: T) ratio was 4:1. K562-GFP target cells were gated by GFP expression, and the dead K562-GFP cells were indicated by near–infrared (IR) staining. K562-GFP without effector cells was negative control and K562-GFP co-cultured with wide type IMC-1 (without transfected any shRNA) was positive control. One representative experiment was shown (n=3).


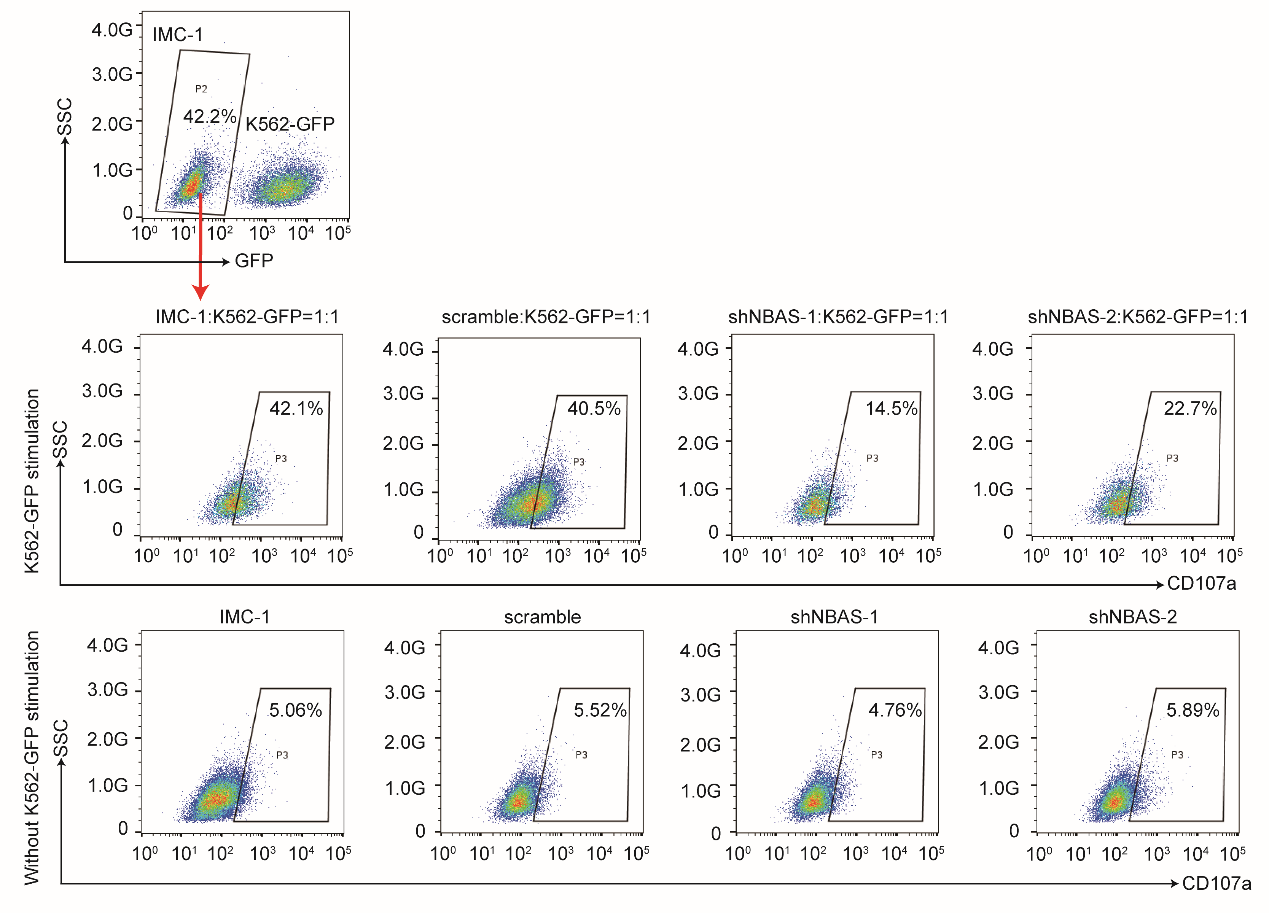


**Fig. S5. Knockdown NBAS impairs the degranulation capacity in NK cell line.** Flow cytometric analysis of degranulation of scramble or shNBAS IMC-1 cells indicated by the expression of surface CD107a. Scramble or shNBAS IMC-1 effector cells were simulated by K562-GFP target cells for 4 hours. The effector-to-target (E: T) ratio was 1:1. IMC-1 cells were gated and the expressions of CD107a were detected by flow cytometry. Wide type IMC-1 cells (without transfected any shRNA) without K562-GFP stimulation were negative control and wide type IMC-1 cells (without transfected any shRNA) stimulated with K562-GFP were positive control. One representative experiment was shown (n=3).


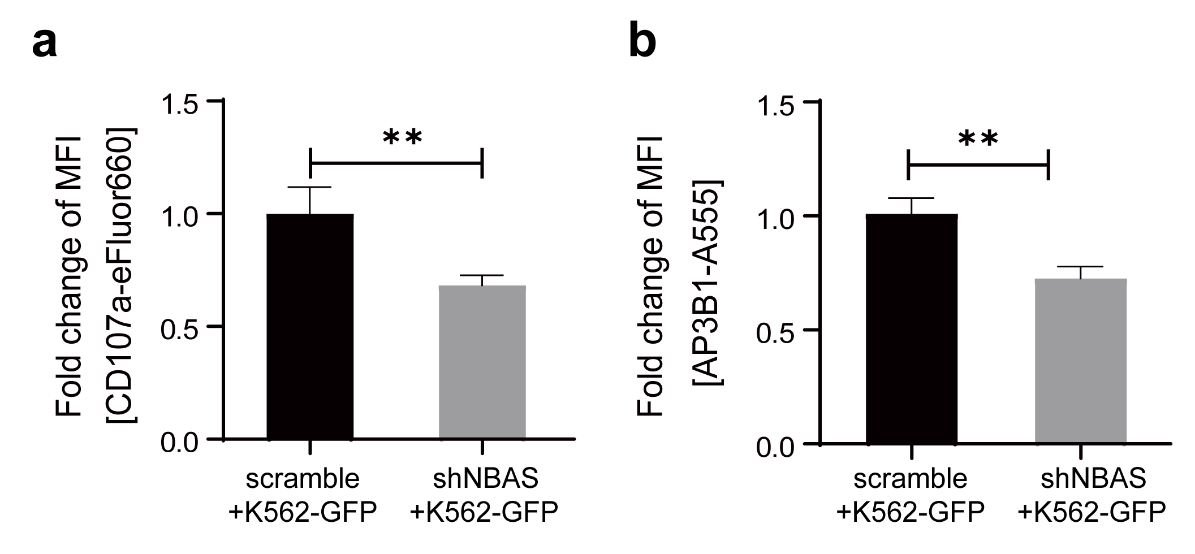


**Fig. S6.** The relative expression of CD107a (red; indicated by yellow arrows) **(a)** and AP3B1 (magenta; indicated by white arrows) **(b)** in Fig. 2i were quantified by mean fluorescence intensity (MFI). The MFI was labeled in the upper-left of Fig. 2i. Wild type IMC-1 (without transfected any shRNA), K562-GFP and wild type IMC-1 co-cultured with K562-GFP as negative controls. Histograms showed the fold change of MFI, after removing the background noise. Shown are mean ± SEM (n=3). **P < 0.01, ***P <0.001.

**Reference**

1. Henter JI, Horne A, Arico M, Egeler RM, Filipovich AH, Imashuku S et al. HLH-2004: Diagnostic and therapeutic guidelines for hemophagocytic lymphohistiocytosis. Pediatr Blood Cancer. 2007;48(2):124-31. doi:10.1002/pbc.21039.

2. Kircher M, Witten DM, Jain P, O'Roak BJ, Cooper GM, Shendure J. A general framework for estimating the relative pathogenicity of human genetic variants. Nat Genet. 2014;46(3):310-5. doi:10.1038/ng.2892.

3. Adzhubei IA, Schmidt S, Peshkin L, Ramensky VE, Gerasimova A, Bork P et al. A method and server for predicting damaging missense mutations. Nat Methods. 2010;7(4):248-9. doi:10.1038/nmeth0410-248.

4. Du Z, Ma L, Qu H, Chen W, Zhang B, Lu X et al. Whole Genome Analyses of Chinese Population and De Novo Assembly of A Northern Han Genome. Genomics Proteomics Bioinformatics. 2019;17(3):229-47. doi:10.1016/j.gpb.2019.07.002.

5. Vazquez A, Flammini A, Maritan A, Vespignani A. Global protein function prediction from protein-protein interaction networks. Nat Biotechnol. 2003;21(6):697-700. doi:10.1038/nbt825.

6. Wang X, Gulbahce N, Yu H. Network-based methods for human disease gene prediction. Brief Funct Genomics. 2011;10(5):280-93. doi:10.1093/bfgp/elr024.

7. Xu J, Li Y. Discovering disease-genes by topological features in human protein-protein interaction network. Bioinformatics. 2006;22(22):2800-5. doi:10.1093/bioinformatics/btl467.

8. Shannon P, Markiel A, Ozier O, Baliga NS, Wang JT, Ramage D et al. Cytoscape: a software environment for integrated models of biomolecular interaction networks. Genome Res. 2003;13(11):2498-504. doi:10.1101/gr.1239303.

9. Schulert GS, Zhang M, Fall N, Husami A, Kissell D, Hanosh A et al. Whole-Exome Sequencing Reveals Mutations in Genes Linked to Hemophagocytic Lymphohistiocytosis and Macrophage Activation Syndrome in Fatal Cases of H1N1 Influenza. J Infect Dis. 2016;213(7):1180-8. doi:10.1093/infdis/jiv550.

10. Kantakamalakul W, Jaroenpool J, Pattanapanyasat K. A novel enhanced green fluorescent protein (EGFP)-K562 flow cytometric method for measuring natural killer (NK) cell cytotoxic activity. J Immunol Methods. 2003;272(1-2):189-97. doi:10.1016/s0022-1759(02)00505-7.

11. Bryceson YT, Fauriat C, Nunes JM, Wood SM, Bjorkstrom NK, Long EO et al. Functional analysis of human NK cells by flow cytometry. Methods Mol Biol. 2010;612:335-52. doi:10.1007/978-1-60761-362-6_23.

12. Wang Y, Song F, Zhu J, Zhang S, Yang Y, Chen T et al. GSA: Genome Sequence Archive<sup/>. Genomics Proteomics Bioinformatics. 2017;15(1):14-8. doi:10.1016/j.gpb.2017.01.001.

13. National Genomics Data Center M, Partners. Database Resources of the National Genomics Data Center in 2020. Nucleic Acids Res. 2020;48(D1):D24-D33. doi:10.1093/nar/gkz913.
